# Opium and Religion

**Published:** 1875-01

**Authors:** 


### OPIUM AND RELIGION.

No opium smoker can be received as a member of a Christian church in China. If it were permitted, it would be a disgrace to the religion of the Bible, a hissing and a by-word in the estimation of the few Chinese Christians. Missionaries are constantly taunted with the charge that foreigners have forced the nation to admit opium into its ports. This seems to Americans one of the greatest national disgraces of England, in compelling the Chinese nation to permit the introduction of cargoes of opium, which the lower classes obtain, as the lower classes in this country obtain liquor and use it to their own destruction.

Americans regard the Chinese as a kind of barbarous people, and yet that government, many years ago, seeing the misery which the use of opium caused to multitudes, determined, as a measure of self-protection, to prohibit the introduction of the drug into the country, as the shortest means of remedying the dreadful evils which its employment induced. But the trade in opium was so profitable to British ships and British subjects that an English fleet was sent to enforce the abrogation of the law, just as the French a few years ago compelled the Sandwich Islands to receive their ships freighted with brandy, and to encourage prostitution with French sailors—thus introducing diseases which are now destroying the native population to such an extent that in a few years it will become an extinct people. The Methodists, in some parts of our country, are making an effort to exclude tobacco-users from church membership; and more generally, to refuse permission to preach to any man who smokes, chews, or snuffs.

Whether the lines are to be more closely drawn around Christian churches in the near future; or whether national virtue is to rise to the height of putting it out of the power of the people to become the victims of any form of intemperance, are questions to be decided in the future.

